# Evaluation of a kDNA-Based qPCR Assay for the Detection and Quantification of Old World *Leishmania* Species

**DOI:** 10.3390/microorganisms8122006

**Published:** 2020-12-16

**Authors:** Marcello Ceccarelli, Gloria Buffi, Aurora Diotallevi, Francesca Andreoni, Daniela Bencardino, Fabrizio Vitale, Germano Castelli, Federica Bruno, Mauro Magnani, Luca Galluzzi

**Affiliations:** 1 Department of Biomolecular Sciences, University of Urbino “Carlo Bo”, 61029 Urbino, PU, Italy; m.ceccarelli3@campus.uniurb.it (M.C.); g.buffi@campus.uniurb.it (G.B.); aurora.diotallevi@uniurb.it (A.D.); francesca.andreoni@uniurb.it (F.A.); daniela.bencardino@uniurb.it (D.B.); mauro.magnani@uniurb.it (M.M.); 2 National Reference Center for Leishmaniasis (C.Re.Na.L.), Istituto Zooprofilattico Sperimentale della Sicilia, 90129 Palermo, PU, Italy; fabrizio.vitale@izssicilia.it (F.V.); germanocastelli@gmail.com (G.C.); federica.bruno@izssicilia.it (F.B.)

**Keywords:** *Leishmania*, *Viannia*, qPCR, Old World, high resolution melting, kDNA

## Abstract

The parasite protozoan *Leishmania*, the causative agent of leishmaniasis, includes two subgenera of medical interest: *Leishmania* (*Leishmania*) and *Leishmania* (*Viannia*). Parasite species detection and characterization is crucial to choose treatment protocols and to monitor the disease evolution. Molecular approaches can speed up and simplify the diagnostic process. In particular, several molecular assays target the mitochondrial DNA minicircle network (kDNA) that characterizes the *Leishmania* genus. We previously proposed a qPCR assay targeting kDNA, followed by high resolution melt (HRM) analysis (qPCR-ML) to distinguish *L.* (*L*.) *infantum* and *L.* (*L*.) *amazonensis* from *L. Viannia* species. Successively, this assay has been integrated with other qPCR assays, to differentiate *L.* (*L*.) *infantum*, *L.* (*L*.) *amazonensis* and *L.* (*L*.) *mexicana.* In this work, we tested the applicability of our qPCR-ML assay on *L.* (*L*.) *donovani*, *L.* (*L*.) *major*, *L.* (*L*.) *tropica* and *L.* (*L*.) *aethiopica*, showing that the qPCR-ML assay can also amplify Old World species, different from *L.* (*L*.) *infantum*, with good quantification limits (1 × 10^−4^–1 × 10^−6^ ng/pcr tube). Moreover, we evaluated 11 *L.* (*L*.) *infantum* strains/isolates, evidencing the variability of the kDNA minicircle target molecules among the strains/isolates of the same species, and pointing out the possibility of quantification using different strains as reference. Taken together, these data account for the consideration of qPCR-ML as a quantitative pan-*Leishmania* assay.

## 1. Introduction

*Leishmania* spp. is a protozoan parasite widespread in Asia, Africa, Europe and America. The parasite is the etiological agent of leishmaniasis, one of the most important tropical diseases that encompasses a broad spectrum of clinical manifestations. The genus *Leishmania* ascribes two subgenera of medical interest: *Leishmania* (*Leishmania*) and *Leishmania* (*Viannia*) [1]. The subgenus *Leishmania* (*Leishmania*) includes species responsible for visceral leishmaniasis (VL) [*L.* (*L*.) *donovani*, *L.* (*L*.) *infantum*] and cutaneous leishmaniasis (CL) [*L.* (*L*.) *infantum*, *L.* (*L*.) *major*, *L.* (*L*.) *tropica*, *L.* (*L*.) *aethiopica*, *L.* (*L*.) *mexicana*, *L.* (*L*.) *amazonensis*], while the species belonging to the subgenus *Leishmania* (*Viannia*) are the etiological agents of CL and mucocutaneous leishmaniasis (MCL) [2]. However, a few *Leishmania* (*Leishmania*) species [i.e., *L.* (*L*.) *infantum*, *L.* (*L*.) *major* and *L.* (*L*.) *tropica*] have also been reported to be the etiological agent of MCL [3]. The diagnosis of leishmaniasis is based on a combination of clinical manifestations, epidemiological data and laboratory test (i.e., serological, parasitological or molecular methods). However, a real gold-standard method for human or veterinary leishmaniasis is still lacking [4]. Among the diagnostic methods, molecular assays allow to obtain fast results and high sensitivity. In particular, a number of PCR-based assays are targeting the mitochondrial DNA minicircle network (kDNA) that characterizes the *Leishmania* genus, ensuring very high sensitivity [5]. Previously, we proposed a qPCR assay targeting the conserved region of kDNA minicircles using primers MLF and MLR (qPCR-ML), followed by high resolution melt (HRM) analysis to detect and distinguish *L*. (*Leishmania*) from *L.* (*Viannia*) subgenus [6]. Successively, this assay has been integrated with other qPCR assays to differentiate *L.* (*L*.) *infantum* from *L.* (*L*.) *amazonensis* [7] and from *L.* (*L*.) *mexicana* [8]. These approaches have also been applied to Brazilian clinical samples, allowing to distinguish different species causing infection in the New World [9]. However, considering the Old World *Leishmania* species, the qPCR-ML assay has been tested only on *L.* (*L*.) *infantum*. In this work, we tested the applicability of our qPCR-ML assay in Old World *Leishmania* species different from *L.* (*L*.) *infantum* [i.e., *L.* (*L*.) *donovani*, *L.* (*L*.) *major*, *L.* (*L*.) *tropica* and *L.* (*L*.) *aethiopica*], in an attempt to test the pan-*Leishmania* potential of our approach. Moreover, we quantitatively evaluated the target sequence of qPCR-ML in different *L.* (*L*.) *infantum* strains and isolates to investigate the possibility of quantification in clinical applications.

## 2. Materials and Methods

### 2.1. Leishmania Strains, Clinical Isolates and Clinical Samples

*Leishmania* strains or isolates used in this study are listed in Table 1. The DNA from all strains and isolates was Chelex-purified from cultured promastigotes and was provided by the Istituto Zooprofilattico Sperimentale della Sicilia, OIE Reference Laboratory, National Reference Center for Leishmaniasis (C.Re.Na.L.), (Palermo, Italy), with the following exceptions: *L.* (*L*.) *donovani* MHOM/IN/02/BPK282/0cl4, provided by the Istituto Zooprofilattico Sperimentale della Lombardia e dell’Emilia-Romagna (Modena, Italy); *L.* (*V*.) *braziliensis* MHOM/BR/1987/M11272, provided by Universidade Federal da Grande Dourados (Brazil); *L.* (*L*.) *amazonensis* MHOM/BR/1973/M2269, *L.* (*L*.) *donovani* MHOM/SD/62/1S, *L.* (*L.*) *major* SV39 and *L. (S.) tarentolae* Parrot Tar II, provided by Instituto de Investigação e Inovação em Saúde Universidade do Porto, Portugal. Moreover, the *L.* (*V*.) *braziliensis* clinical isolate AN1 was obtained from a pharyngo-laryngeal biopsy during routine diagnosis of a human patient with MCL. The human clinical samples Psalb and Daedio were obtained during routine diagnosis of two patients with VL (blood) [10] and CL (cutaneous biopsy), respectively. The DNA samples were quantified using a Qubit fluorometer 3 (Thermofisher, Carlsbad, CA, USA).

### 2.2. qPCR Assay

The qPCR assays were performed using MLF-MLR primer pair (MLF: 5′-CGTTCTGCGAAAACCGAAA-3′; MLR: 5′-CGGCCCTATTTTACACCAACC-3′) as previously reported [6]. Briefly, the reactions were carried out in 25 μL volume with 1 μL of template DNA and 24 μL TB Green Premix Ex TaqII Mastermix (Takara Bio Europe, France) containing 200 nM MLF and MLR primers. The reactions were performed in a Rotor-Gene 6000 instrument (Corbett Life Science, Mortlake, Australia). The cycle threshold (Cq) values were determined using the quantitation analysis of the Rotor-Gene 6000 software, setting a threshold to 0.15.

Standard curves were established using 1 × 10^0^ to 1 × 10^−7^ ng DNA of *L.* (*L*.) *major* MHOM/SU/73/5ASKH, *L.* (*L*.) *donovani* MHOM/IN/80/DD8, *L.* (*L*.) *aethiopica* MHOM/ET/72/L100, *L.* (*L*.) *tropica* MHOM/SU/74/K27, *L.* (*L*.) *infantum* MHOM/FR/78/LEM75 and *L. (S.) tarentolae* Parrot Tar II. To evaluate the potential interference of host DNA as background in the qPCR assay, 30 ng of human DNA purified from human cell line (CACO-2 or MCF7) was spiked in each qPCR reaction tube (with the exception of *L. (S.) tarentolae* Parrot Tar II because not pathogenic to humans). The standard curves were obtained from two independent experiments for each strain, processed in duplicate.

To determine the variability of kDNA minicircle target sequences in *L.* (*L*.) *infantum* species, 0.02 ng DNA extracted from 11 *L.* (*L*.) *infantum* strains/isolates were amplified in 6 independent experiments and Cq were compared by Kruskal–Wallis nonparametric test.

### 2.3. High-Resolution Melt (HRM) Analysis

The qPCR-ML amplicons, obtained by all stains/isolates listed in Table 1, were analyzed by HRM protocol on a Rotor-Gene 6000 instrument as described previously [6] with slight modifications. Briefly, HRM was carried out over the range from 77 °C to 89 °C, rising at 0.1 °C/s and waiting for 2 s at each temperature. Each sample was run in duplicate and the gain was optimized before melting on all tubes. HRM curve analysis was performed with the derivative of the raw data, after smoothing, with the Rotor-Gene 6000 software. Only the samples with Cq values ˂ 30 were considered [11]. The HRM temperature average and the standard deviation derived from 3 independent experiments.

### 2.4. PCR Product Sequencing

The qPCR-ML products obtained from *L.* (*L*.) *major* MHOM/SU/73/5ASKH, *L.* (*L*.) *major* MRHO/SU/59/P-strain, *L.* (*L*.) *donovani* MHOM/IN/80/DD8, *L.* (*L*.) *aethiopica* MHOM/ET/72/L100, *L.* (*L*.) *tropica* MHOM/SU/74/K27, *L.* (*L*.) *infantum* MHOM/FR/78/LEM75, *L.* (*V*.) *braziliensis* MHOM/BR/75/M2904 were purified using the MinElute PCR purification kit (Qiagen) and directly sequenced, using both MLF and MLR primers, as previously described [6,7]. The DNA sequencing was performed using the BigDye Terminator v. 1.1 Cycle Sequencing Kit on ABI PRISM 310 Genetic Analyzer (Applied Biosystems, Foster City, CA, USA). Sequences were manually edited and nucleotide composition analyses were conducted using MEGA6 [12]. A phylogenetic tree based on the Maximum Likelihood method was constructed with 10,000 bootstrap replications, using the nearest neighbor interchange method in MEGA6 [12].

### 2.5. Statistical Analysis

To compare the variability on Cq values on *L.* (*L*.) *infantum* strains/isolates, a Kruskal–Wallis nonparametric test followed by Dunn’s multiple-comparison post-test was performed. To evaluate the different HRM temperatures between the *Leishmania* and *Viannia* subgenera, a Mann–Whitney test was performed. All statistical tests were performed using GraphPad Prism version 5 (GraphPad Software, Inc., La Jolla, CA, USA). A *p* value ≤ 0.05 was considered statistically significant.

## 3. Results

### 3.1. The qPCR-ML Assay Can Amplify All Medically Relevant Old World Leishmania Species

**Figure 1:**
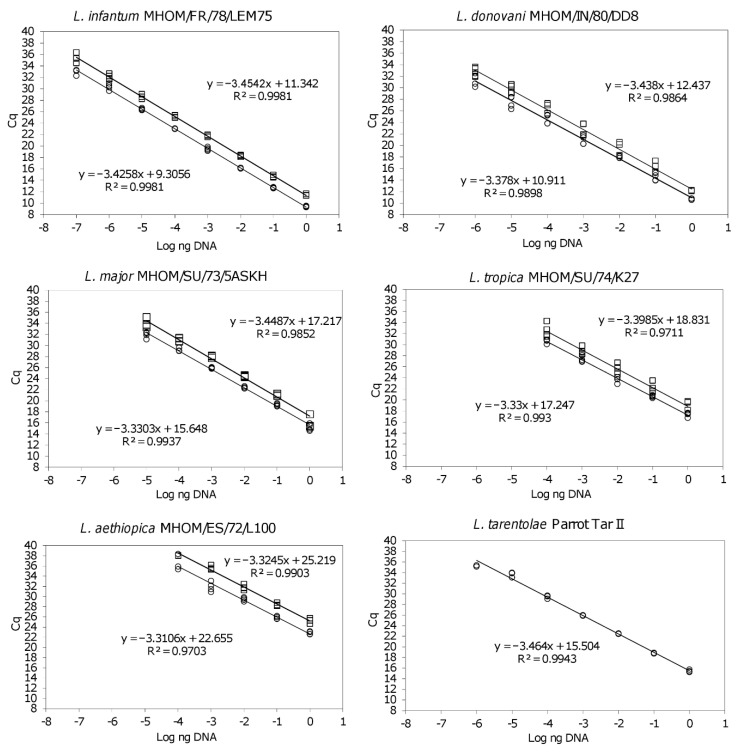
qPCR-ML standard curves obtained from DNA serial dilutions of *L.* (*L*.) *infantum* MHOM/FR/78/LEM75, *L.* (*L*.) *donovani* MHOM/IN/80/DD8, *L.* (*L*.) *major* MHOM/SU/73/5ASKH, *L.* (*L*.) *tropica* MHOM/SU/74/K27, *L.* (*L*.) *aethiopica* MHOM/ET/72/L100, *L. (S.) tarentolae* Parrot Tar II. The standard curves were spiked (upper curve, square points) or non-spiked (lower curve, circle points) with 30 ng human DNA. The standard curve of *L. (S.) tarentolae* Parrot Tar II, not pathogenic to humans, was not spiked. Each point derived from a duplicate of two independent experiments.

The qPCR-ML was applied to the strains listed in Table 1 to verify the amplifiability of all *Leishmania* species with MLF-MLR primers. All Old World species [i.e., *L.* (*L*.) *infantum*, *L.* (*L*.) *donovani*, *L.* (*L*.) *major*, *L.* (*L*.) *tropica*, *L.* (*L*.) *aethiopica*, *L. (S.) tarentolae*] were successfully amplified. The calibration curves evidenced a limit of quantification of 1 × 10^−4^ ng/PCR tube for *L.* (*L*.) *tropica* and *L.* (*L*.) *aethiopica*, 1 × 10^−5^ ng/PCR tube for *L.* (*L*.) *major*, 1 × 10^−6^ ng/PCR tube for *L.* (*L*.) *donovani*, 1 × 10^−7^ ng/PCR tube for *L.* (*L*.) *infantum* and 1 × 10^−6^ ng/PCR tube for *L. (S.) tarentolae* Parrot Tar II, with PCR efficiencies ≥ 94.5% and R^2^ ˃ 0.97 (Figure 1). Although out of linearity range, the limit of detection for *L.* (*L*.) *donovani* MHOM/IN/80/DD8 and *L.* (*L*.) *tropica* MHOM/SU/74/K27 were 1 × 10^−7^ (Cq 32.48 ± 0.46) and 1 × 10^−5^ (Cq 33.56 ± 1.14) ng/PCR tube, respectively. In the presence of human DNA as background, the Cq were slightly delayed, but the linearity and limit of quantification of the assay remained unchanged (Figure 1).

**Figure 2:**
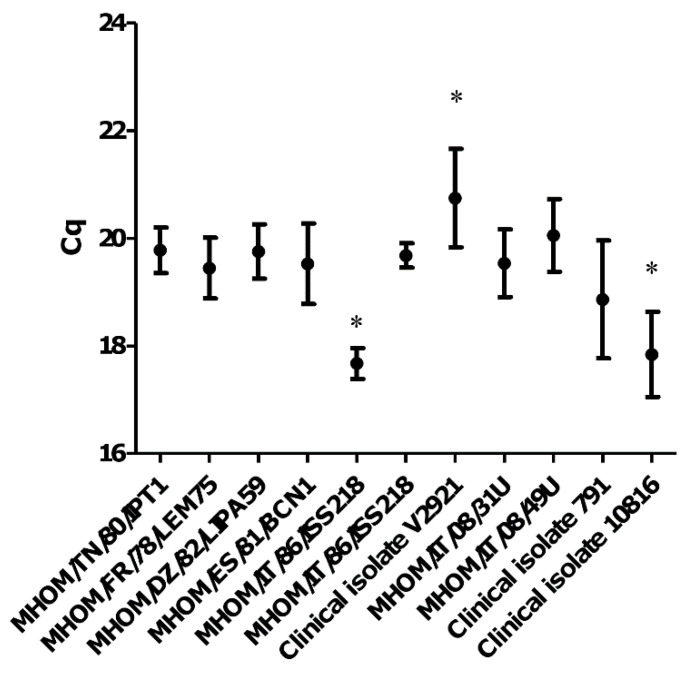
qPCR-ML Cq values obtained from 11 *L.* (*L*.) *infantum* strains/isolates. The results were obtained from six independent experiments. * *p* < 0.01 Dunn’s Multiple Comparison Test.

The variability of kDNA minicircle target sequence was investigated among 11 *L.* (*L*.) *infantum* strains by amplification of 0.02 ng promastigote DNA. Dunn’s post-test analysis of Cq values revealed significant differences among V2921 clinical isolate, MHOM/IT/86/ISS218 strain and clinical isolate 10816 (Figure 2). The average Cq was 19.36 ± 1.14, very close to the value obtained for the strain MHOM/FR/78/LEM75 (19.44 ± 0.56), suggesting that this strain could be used as reference to obtain approximate quantification values for illness caused by *L.* (*L*.) *infantum*.

### 3.2. The subgenera Leishmania and Viannia Can Be Differentiated by HRM Analysis

**Figure 3:**
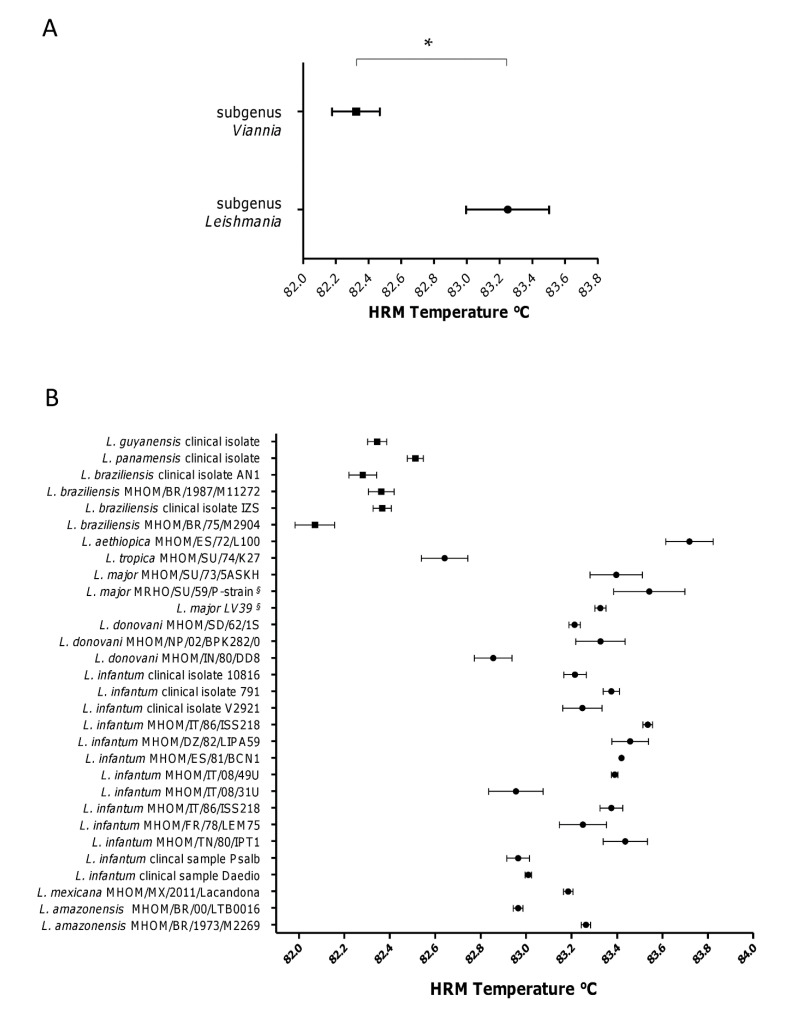
*Leishmania* spp. qPCR-ML HRM temperatures analysis. (**A**) Comparison between HRM temperatures (mean and standard deviation) of all tested samples belonging to subgenus *Leishmania* (n = 24) and *Viannia* (n = 6). * *p* < 0.05 Mann–Whitney test. (**B**) HRM temperature distribution of all samples tested. Squares and circles represent species belonging to subgenus *Viannia* and *Leishmania*, respectively. Mean values and standard deviations of at least three replicates are shown. ^§^ synonym strains coming from different laboratories.

Species discrimination power of qPCR-ML assay was also evaluated by analysis of HRM profiles on all *Leishmania* species listed in Table 1. Despite the impossibility to reliably discriminate each species using HRM profiles, the assay evidenced the lower Tm of amplicons belonging to *Viannia* subgenus, confirming the possibility to discriminate between *Leishmania* and *Viannia* subgenus, as previously demonstrated with a limited number of species [6] (Figure 3A). However, the Tm of *L.* (*L*.) *tropica* MHOM/SU/74/K27 amplicon showed a partial overlapping with *L.* (*V*.) *panamensis*, hence the two species were not fully distinguishable (Figure 3B). Accordingly, the specificity of this assay can be considered 96.6%. Nevertheless, this drawback can be overcome by considering the geographical origin of the sample since *L.* (*L*.) *tropica* is an Old World species. It is also noteworthy that *L. (S.) tarentolae* Parrot Tar II HRM temperature was 80.92 ± 0.08, clearly distinguishable from *Viannia* and *Leishmania* subgenera.

The sequence analysis of qPCR-ML products evidenced that the GC content found in *L. Viannia* strains was lower than that found in *L. Leishmania* strains (<48.2% and ˃48.6%, respectively), accounting for the lower Tm observed in the *Viannia* amplicons. In addition, a maximum likelihood phylogenetic tree of qPCR-ML amplicons was performed, confirming the clusterization in two groups of the amplicons obtained from the two subgenera, with the exception of *L.* (*L*.) *tropica*, which clusterizes independently (Figure S1).

## 4. Discussion

Although leishmaniasis is considered a neglected disease, it affects about 12 million people in Africa, Americas, Asia and Europe, with a heterogeneous clinical spectrum [13]. The diagnosis is complicated, as well as for the different species spread in the world. To improve the diagnostic process and classification, a number of molecular assays have been developed. In particular, qPCR techniques allow obtaining rapid parasitic load estimation and potential species discrimination through melting analysis. Due to their high copy number per cell, the kDNA minicircles are considered molecular targets that allow to design very sensitive assays [14]. However, due to the different composition of minicircle classes in the various species, an assay designed on a particular species can show lower sensitivity in another [7]. For this reason, many works describe minicircle-based qPCR assays targeting only few species or complex. For example, some authors developed qPCR assay for New World species [15,16], while some others tested their primers in Old World species [17,18]. Since different reports describe the infection in travelers [19,20] and in military personnel operating in country endemic for leishmaniasis [21,22,23], the availability of a rapid pan*-Leishmania* assay, highly sensitive and able to provide a quantification and a partial classification, can certainly be useful. Moreover, a pan-*Leishmania* assay can also be exploited in eco-epidemiological studies, including identification of vectors and reservoirs [24]. In recent years, only a few attempts to develop pan-*Leishmania* assays based on nucleic acids amplification (i.e., SL RNA, rDNA or kDNA minicircles) have been made [24,25,26,27]. We previously showed that qPCR-ML was able to detect Old World *L.* (*L*.) *infantum* and New world species [i.e., *L.* (*L.*) *chagasi/infantum*, *L.* (*L*.) *amazonensis*, *L.* (*L*.) *mexicana*, *L.* (*V*.) *guyanensis*, *L.* (*V*.) *panamensis*, *L.* (*V*.) *braziliensis*] and among these species, distinguishing between *Leishmania* and *Viannia* subgenera [6,8]. In this work, we show for the first time that the qPCR-ML assay can also amplify the Old World species different from *L.* (*L*.) *infantum* with good quantification limits (1 × 10^−4^–1 × 10^−6^ ng/pcr tube), including *L. (S.) tarentolae*, not pathogenic to humans. The differences in the limit of quantification of the different species can be explained by different amount of minicircle class amplified by ML primers, as already shown for other species [7]. Importantly, we confirmed that species related to *Viannia* subgenus can be distinguished from Old World species of *Leishmania* subgenus by HRM analysis, with the only exception of *L.* (*L*.) *tropica* that, however, is distinguishable from *Viannia* subgenus by phylogenetic analysis. Moreover, although caution should be taken due to the few strains tested, it is interesting to note that the related species *L.* (*L*.) *tropica* and *L.* (*L*.) *aethiopica* can be distinguished through HRM analysis (Figure 3). *L.* (*L*.) *aethiopica* is geographically restricted to Kenya and Ethiopia, while *L.* (*L*.) *tropica* is widely distributed from the Eastern Mediterranean to Eastern India and in Africa. These species were distinguished by microsatellite analysis [28] but, to our knowledge, this is the first evidence of their separation using an HRM-based approach. In fact, *L.* (*L*.) *aethiopica* is a species rarely tested using HRM analysis and, when done, it is without discrimination from other species [29]. On the whole, due to its good sensitivity especially in Old World species, the qPCR-ML assay appears to be applicable in human and veterinary medicine in Europe, Africa and Middle East. Similar approaches having ITS1 region as target have been previously published [30,31]. Although these ITS1-based assays have the great potential to discriminate among *Leishmania Leishmania* species through HRM analysis [30], their contextual application with the *Viannia* subgenus has not been reported. Moreover, the use of FRET probes for species discrimination [31] could increase the cost compared to approaches based on intercalating dyes. The lack of species specificity of the qPCR-ML assay can be overcome using sequential PCR assays, as already demonstrated [8] or, in part, with the contextualization between the clinical presentation and the geographical origin of the patient.

Finally, we quantitatively evaluated the minicircle subclass amplified by qPCR-ML in 11 different *L.* (*L*.) *infantum* strains/isolates, to verify the possible differences in terms of Cq values. We confirmed the presence of significant variability among some strains/isolates, as previously reported in literature [14,32]. This variation could have an impact on absolute quantification, but the use of a reference strain with average minicircle kDNA amplifiable sequences could minimize the variability of results. Moreover, it could have a negligible consequence on the biological and clinical interpretation of diagnostic results, as already pointed out by Mary et al. [14].

## 5. Conclusions

In conclusion, the present work represents an update of our assay mentioned above and it demonstrates the applicability of qPCR-ML to detect and to quantify a large spectrum of *Leishmania* species, including also the Old World species, with a good limit of quantification and detection. We believe that this assay could be now considered as a quantitative pan-*Leishmania* assay, because it has been tested in the most common *Leishmania* species of the New and Old World, including *L. (S.) tarentolae*.

## Figures and Tables

**Table 1:** *Leishmania* spp. reference strains, clinical isolates and clinical samples used in this study.

Subgenus	Species	Strain/Isolate
*Leishmania*	*L. infantum*	MHOM/TN/80/IPT1
*Leishmania*	*L. infantum*	MHOM/FR/78/LEM75
*Leishmania*	*L. infantum*	Clinical isolate V2921
*Leishmania*	*L. infantum*	MHOM/IT/08/31U
*Leishmania*	*L. infantum*	MHOM/IT/08/49U
*Leishmania*	*L. infantum*	Clinical isolate 10816
*Leishmania*	*L. infantum*	Clinical isolate 791
*Leishmania*	*L. infantum*	MHOM/DZ/82/LIPA59
*Leishmania*	*L. infantum*	MHOM/ES/81/BCN1
*Leishmania*	*L. infantum*	MHOM/IT/86/ISS218
*Leishmania*	*L. infantum*	MHOM/IT/93/ISS822
*Leishmania*	*L. donovani*	MHOM/IN/80/DD8
*Leishmania*	*L. donovani*	MHOM/NP/02/BPK282/0cl4
*Leishmania*	*L. donovani*	MHOM/SD/62/1S
*Leishmania*	*L. major*	MHOM/SU/73/5ASKH
*Leishmania*	*L. major*	MRHO/SU/59/P-strain *
*Leishmania*	*L. major*	SV39 *
*Leishmania*	*L. tropica*	MHOM/SU/74/K27
*Leishmania*	*L. aethiopica*	MHOM/ET/72/L100
*Leishmania*	*L. amazonensis*	MHOM/BR/00/LTB0016
*Leishmania*	*L. amazonensis*	MHOM/BR/1973/M2269
*Leishmania*	*L. mexicana*	MHOM/MX/2011/Lacandona
*Leishmania*	*L. infantum*	Clinical sample Psalb
*Leishmania*	*L. infantum*	Clinical sample Daedio
*Viannia*	*L. panamensis*	Clinical isolate
*Viannia*	*L. guyanensis*	Clinical isolate
*Viannia*	*L. braziliensis*	Clinical isolate AN1
*Viannia*	*L. braziliensis*	Clinical isolate IZS
*Viannia*	*L. braziliensis*	MHOM/BR/1987/M11272
*Viannia*	*L. braziliensis*	MHOM/BR/75/M2904
*Sauroleishmania*	*L. tarentolae*	Parrot Tar II

* synonym strains coming from two different laboratories.

## Supplementary Materials

The following are available online at https://www.mdpi.com/2076-2607/8/12/2006/s1, Figure S1: Maximum likelihood phylogenetic tree of qPCR-ML amplicons.
