# Supplementary material for: Evaluation of a kDNA-Based qPCR Assay for the Detection and Quantification of Old World Leishmania Species
Source: Microorganisms. 2020 Dec 16;8(12):2006. doi: 10.3390/microorganisms8122006 (PMC7765608; doi:10.3390/microorganisms8122006)
Supplement: Supplementary file 1 [file microorganisms-08-02006-s001.zip › Figure S1.pdf]

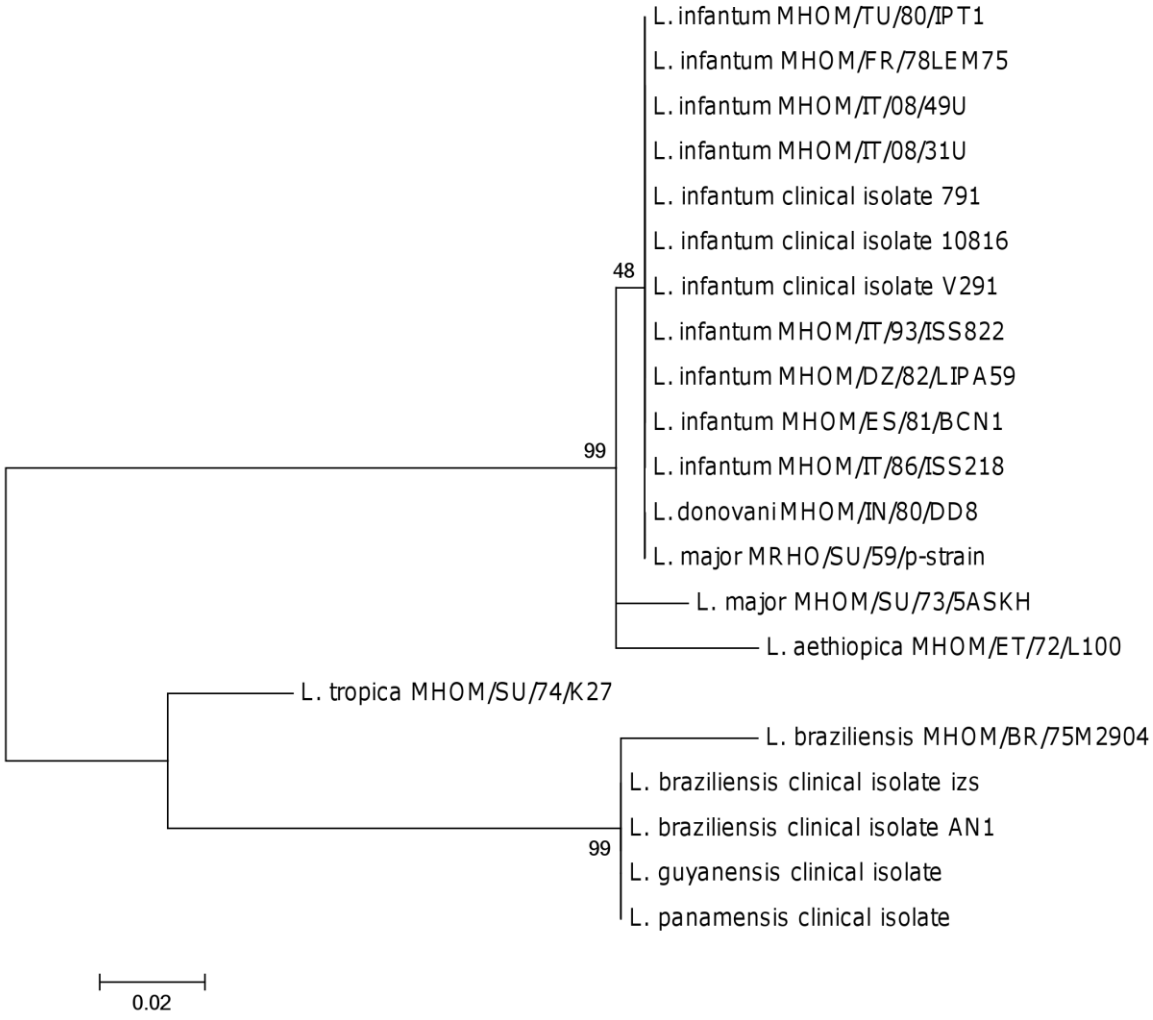

Figure S1. Maximum likelihood phylogenetic tree of qPCR-ML amplicons. The tree was constructed using the nearest neighbor interchange method with MEGA 6.0 software.
